# Attitudes of polish nurses towards representatives of certain religions

**DOI:** 10.1186/s12912-021-00798-7

**Published:** 2022-01-20

**Authors:** Joanna Zalewska-Puchała, Iwona Bodys-Cupak, Anna Majda

**Affiliations:** Theory and Fundamentals of Nursing Laboratory, Institute of Nursing and Midwifery, Faculty of Health Sciences, Jagiellonian University Medical College, Krakow, Poland

**Keywords:** Nurses, Social distance, Religion

## Abstract

**Background:**

The verification of attitudes of nurses towards people of different religions is essential when it comes to anticipating opportunities for cooperation in the field of provision of healthcare. The purpose of the study was to evaluate the attitudes of Polish nurses, on the example of nurses living in the Lesser Poland region (southern Poland) towards representatives of certain religions.

**Methods:**

The study was cross-sectional and was carried out using both the diagnostic survey and method of estimation. The selection of the study group was purposeful. The research tools used in the study include the Bogardus’ Social Distance Scale adapted by Jasińska-Kania and Staszyńska; Social Dominance Orientation (SDO) by Sidanius and Pratto; Social Approbation Questionnaire by Drwal and Wilczyńska and Author’s questionnaire. A significance level of 0.05 was assumed in the analyzes. The research was carried among 1271 professionally active nurses.

**Results:**

The respondents showed the least significant social distance towards the Orthodox and Protestants and the most significant towards the Muslims. There was a considerable dependence when it comes to the level of the social distance of the respondents shown on sociodemographic variables and frequency of contact with Others, provision of healthcare to people of different religions, and training on transcultural nursing. The relationship between SDO and the modified Bogardus Scale has been shown. All subscales of the Social Dominance Orientation (SDO) correlated significantly and negatively with the Bogardus scale.

**Conclusions:**

The study outcomes show that there is a need to train nurses on transcultural nursing.

## Background

Polish society is relatively homogeneous when it comes to religion. Apart from Catholics, the adherents of Orthodoxy constitute the largest group of over half a million people. There are rather many representatives of Protestantism, the number of which is estimated at 150,000 people. The most numerous religious groups also include Jehovah’s Witnesses, whose number exceeds 125,000 [1]. Nurses are an occupational group that daily experiences contact with people representing different religious beliefs that perceive health and illness differently. This dissimilarity can cause curiosity, fascination, admiration, but it can also be a source of aversion, fear, frustration, and a feeling of threat. Seeing a representative of different religious beliefs as interesting and valuable conduce interactions based on mutual recognition and respect, aims at cooperation and stimulates. On the other hand, seeing them as unimportant and backward triggers aggressive behaviors, antagonisms, and domination making nursing more difficult [2]. Lack of understanding for being different and lack of basic information about cultural differences make it challenging to communicate with the patient, which is extremely important when it comes to providing healthcare services for a patient [3]. Not knowing the foundations of the doctrines of other religions and different views on ethical matters related to medical activities may lead to conflicts resulting from misunderstanding the intentions of patients. Conflicts between healthcare workers and patients, resulting from differences in attitudes and values determined by religious diversity, require discussion on the scope of this phenomenon and its consequences for healthcare. Thus, the problem of the coexistence of various religious communities should be examined and also analyzed in relation to healthcare. Research on attitudes of nurses towards religious diversity seems to be necessary and empirical analysis-worth. Evaluation of these attitudes is important not only for anticipation of the opportunities for cooperation in terms of healthcare for various communities and the quality of nursing care, but also for noticing the possible risk of mutual conflicts that affect communication, identifying needs and health problems, and consequently, the satisfaction of nursing care by representatives of different religions.

## Methods

### Study design

The study was cross-sectional and was conducted using the diagnostic survey and estimation methods. The reason behind choosing the cross-sectional nature of the study was the fact that it provides results relatively quickly on a large group of respondents. The purpose of the study was to evaluate the attitudes of Polish nurses, on the example of nurses living in the Lesser Poland region (southern Poland) towards representatives of certain religions. Also, it was important in the study to determine to what extent variables such as dominance orientation, culture-based education, work experience and contact with people of different religious beliefs, and selected sociodemographic data can affect nurses’ attitudes towards selected representatives of different religions.

### Setting and participants

The selection of the study group was purposeful. The questionnaires were provided personally to nurses employed in hospitals located in 15 poviats of the Lesser Poland (incl. Bochnia, Brzesko, Chrzanów, Dąbrowa Tarnowska, Gorlice, Kraków, Limanowa, Nowy Sącz, Nowy Targ, Sucha Beskidzka, Olkusz, Proszowice, Tarnów, Wadowice, Zakopane). The total number of nurses employed in the hospitals that participated in the studies was 4663. A total of 2778 questionnaires were distributed among nurses (participation of nurses in the study was equal to 59.6%), 1794 questionnaires were returned (survey resonance equal to 64.57%), out of which 1621 questionnaires were fully completed. Assuming that the results of the scale of attitudes could be affected by the tendency of intentional or unintentional presenting oneself in a good or bad light. In the study, the Questionnaire of Social Approbation (KAS) was used. The KAS was developed to measure the need for social approbation, understood as a personality trait and a tendency to present oneself in a falsely good light. It consists of 29 statements that require “true” or “false” answers from the respondent. Statements describe behaviors and traits with explicit social approbation or disapprobation but do not contain pathological content [4]. Based on the analysis of the Questionnaire of Social Approbation (coefficient alpha for the study - 0.772), 350 people were identified as having a tendency for simulation or dissimulation that could potentially cause the falsification of the results of the Bogardus Scale. It was therefore decided to exclude these respondents from further analysis. Ultimately, 1271 people were included in the analysis of data.

### Measurement tools

The study was conducted as a part of the statutory project *Attitudes of nurses living in Lesser Poland towards culturally different people* (K/ZDS/007098 2017–2019). Some of the results of the above-mentioned statutory project have already been published [5]. The tools used in the studies include the Bogardus Social Distance Scale adopted by Jasińska-Kania and Staszyńska [6]; Social Dominance Orientation (SDO) by Sidanius and Pratto [7]; Questionnaire of Social Approbation (KAS) by Drwal and Wilczyńska [4] and the questionnaire developed by the Authors with questions related to the experiences of nurses having contact with people of different religions, participation in training on transcultural nursing, and social-demographic information. The research tools have been described in detail in the article Zalewska-Puchała J, Bodys-Cupak I, Majda A. Attitudes of Polish Nurses Toward Selected National Groups. J Transcult Nurs. 2020 [5]. The Social Distance Scale developed by Emory Bogardus is a simple but effective research tool that has become a widely used tool and instrument in the study of intergroup relations. It has been called “one of his most famous tools of social psychology in American intellectual history” [8 , p. 391]. A modified scale was used to assess the stigma of adults due to mental illness [9].

### Data collection

The choice of the research area was purposeful, and it was planned to conduct research in the largest hospitals located in the Małopolska Voivodeship. The study was conducted in 2018 among nurses who are professionally active and employed at various positions in various hospital wards. The consent to conduct the research was obtained in 15 hospitals. *The inclusion* in the study required meeting the following criteria: nurses working in hospitals/clinics in Lesser Poland. The participants were provided with all necessary information about the study, and were informed about its purpose, anonymity, the voluntary nature of the participation in the study, and that they may withdraw from participating in the study at any moment. Exclusion criterion: medical education other than a nurse.

### Statistical methods

The quantitative variables analysis was conducted by calculating the mean, standard deviation, median, quartiles, and both the minimum and maximum. The qualitative variables analysis was conducted by calculating the number and percentage of occurrences of each value. The comparison of distance towards people of different religions was conducted with the use of the ANOVA analysis with repeated measurements or the Friedman test. After finding the statistically significant differences, the post hoc analysis was conducted with the Student’s t-tests for matched pairs or Wilcoxon test for matched pairs to identify the statistically significantly different groups. In both cases, the Bonferroni correction was applied. The comparison of the distance for the two groups was conducted using either Student’s t-test or the Mann–Whitney U test. The comparison of attitudes in three or more groups was conducted using either the ANOVA variance analysis or the Kruskal–Wallis H test. After finding the statistically significant differences, the post hoc analysis was conducted using either Fisher’s LSD test or Dunn’s test to identify statistically significantly different groups. Correlations between social distance and quantitative variables were analyzed with the Pearson correlation coefficient (when both had a normal distribution) or Spearman correlation coefficient (otherwise). The strength of the dependency was interpreted with the accordance to the interpretation scheme after: Hinkle et al.: |r| ≥ 0.9 – very strong dependency; 0.7 ≤ |r| < 0.9 – strong dependency; 0.5 ≤ |r| < 0.7 – medium-strong dependency; 0.3 ≤ |r| < .0.5 – weak dependency; |r| < 0.3 – very weak dependency (negligible) [10]. The normality of variable distribution was examined with the use of the Shapiro-Wilk test. The significance level for the analysis was equal to 0.05. The analysis was conducted in the R program, version 3.5.1 [11].

### Ethical considerations

The study was conducted as a part of the statutory project K/ZDS/007098 and obtained the approval of the Bioethics Committee of the Jagiellonian University No. 1072.6120.196.2017 of November 30th, 2017. The study was developed and conducted according to the principles of good scientific practice, the Act of May 10th, 2018, on the protection of personal data, the principles of the Declaration of the Helsinki and Regulation (EU) 2016/679 of the European Parliament and of the Council of April 27th, 2016. Informed consent was obtained from all participants in this study.

## Results

### Characteristics of the study group

Table 1 presents the sociodemographic data of the respondents.

**Table 1:** The sociodemographic data of the respondents

Parameters		The Study Group
Gender	Female	96.70%
	Male	3.30%
Age		21–75 years of age 46.13 ± 10.09 (Me 49)
Work experience in the profession		1–53 years of age 23.68 ± 11.31 (Me 27)
Education	secondary vocational education	43.04%
	higher undergraduate	39.18%
	higher master’s degree	16.68%
	no answer	1.1%
Specialization in nursing		16.44%
The transcultural nursing training		15.50%
Places of residence	village	49.17%
	city < 20 thousand inhabitants	28.09%
	city > 20 thousand inhabitants	20.54%
	no answer	2.2%
Living abroad (in the past)		12.43%
Working abroad as a nurse (in the past)		2.36%
Religion	Catholics	92.29%
	other religious affiliation	0.55%
	(Protestants, Orthodox, Muslim, Greek Catholic)	
	atheists	1.34%
	no answer	5.82%
Religiousness	“deeply religious”	36.74%
	“rather religious”	55.31%
	“difficult to say”	6.45%
	no answer	1.49%
Religious practices	few times a week	15.97%
	once a week	60.11%
	less than once a week	21.95%
	no answer	1.97%
Private or professional contact with representatives of other religions		93.63%
Providing care for people of different religion (most often they were Jehovah’s Witnesses, Orthodox, Protestants, Seventh-day Adventists)		64.99%

#### Bogardus scale analysis

The Bogardus Scale used in the study in a modified version turned out to be an instrument of high reliability for all religion groups that were studied - coefficient alpha from 0.956 (Muslim) to 0.941 (Jehovah’s Witnesses). The purpose of using the Bogardus scale was to assess to which areas of life and to how close the respondents are willing to let the Other person based on their religion. The study included the following groups: Jews, Orthodox, Protestants, SDA, Jehovah’s Witnesses, Muslims, Hindus, and Buddhists. On the Bogardus scale, adapted by the authors for the purposes of this study, it was decided to distinguish five components [6]:

1. Distance in the macro-social public sphere (accepting that Others live on the territory of Poland, they work in Poland and have Polish citizenship)
2. Distance in the micro-social public sphere (accepting that Other is a co-worker, neighbor, employer, and a political representative at the level of local authorities)
3. Distance in the private sphere (accepting the friendship with the Other)
4. Distance in the intimate sphere (accepting that the family member of the respondent is in a relationship with the Other).
5. Biological distance (allowing to accept a blood transfusion from the Other)

Each of the variables was measured on a 5-point Likert scale, from strong acceptance (5 points), through being neutral (3 points), to strong opposition to the presented situation (1 point). Thus, the higher the score on the scale of a certain distance was obtained by the respondent, the more positive attitude towards the representatives of different religions.

The analysis of the obtained data showed that distances towards different religions differed significantly (*p* < 0,05). The post hoc analysis was conducted to answer the question of what exactly these changes were. The outcomes of the analysis show that the smallest social distance respondents showed towards the Orthodox, and then towards the Protestants, SDA, Jews, Jehovah’s Witnesses, and the largest social distance towards Muslims, Buddhists, and Hindus (Table 2).

**Table 2:** Social distance of the surveyed nurses towards certain religious groups

Parameter		Jews	Orthodox	Protestants	SDA	Jehovah’s Witnesses	Muslims	Hindus	Buddhists	p *
Bogardus Scale	M ± SD	3.1 ± 1.01	3.26 ± 0.97	3.21 ± 0.98	3.17 ± 0.99	3.06 ± 1	2.47 ± 1.08	2.85 ± 1.04	2.9 ± 1.04	*p* < 0.001
	Me	3	3.2	3.1	3.1	3	2.4	3	3	
	Q	2.5–3.8	2.7–4	2.7–4	2.6–3.9	2.4–3.8	1.5–3.1	2.1–3.5	2.2–3.6	
Public distance makro	M ± SD	3.22 ± 1.03	3.4 ± 0.99	3.33 ± 1	3.29 ± 1.02	3.21 ± 1.05	2.54 ± 1.17	2.96 ± 1.11	3.03 ± 1.13	*p* < 0.001
	Me	3	3.33	3.33	3.33	3	2.67	3	3	
	Q	2.67–4	3–4	2.83–4	2.67–4	2.67–4	1.67–3.33	2.33–3.67	2.33–4	
Public distance mikro	M ± SD	2.94 ± 1.1	3.14 ± 1.06	3.09 ± 1.07	3.05 ± 1.07	2.94 ± 1.09	2.31 ± 1.12	2.71 ± 1.1	2.78 ± 1.1	*p* < 0.001
	Me	3	3	3	3	3	2	3	3	
	Q	2–3.75	2.5–4	2.5–4	2.5–4	2.25–3.75	1.12–3	2–3.5	2–3.5	
Private distance	M ± SD	3.28 ± 1.18	3.36 ± 1.13	3.32 ± 1.14	3.27 ± 1.15	3.17 ± 1.46	2.62 ± 1.31	2.97 ± 1.23	3.02 ± 1.22	*p* < 0.001
	Me	3	3	3	3	3	3	3	3	
	Q	3–4	3–4	3–4	3–4	2–4	1–4	2–4	2–4	
Intimate distance	M ± SD	2.92 ± 1.23	3.05 ± 1.2	2.98 ± 1.22	2.92 ± 1.21	2.73 ± 1.25	2.27 ± 1.25	2.59 ± 1.25	2.62 ± 1.25	*p* < 0.001
	Me	3	3	3	3	3	2	3	3	
	Q	2–4	2–4	2–4	2–4	2–4	1–3	1–3	1–3	
Biological distance	M ± SD	3.39 ± 1.3	3.46 ± 1.25	3.44 ± 1.25	3.39 ± 1.27	3.31 ± 1.31	2.93 ± 1.43	3.16 ± 1.34	3.19 ± 1.32	*p* < 0.001
	Me	4	4	4	4	3	3	3	3	
	Q	3–4	3–4	3–4	3–4	3–4	2–4	2–4	2–4	

* NP = Lack of distribution of normality for at least one religion, Friedman’s test + post-hoc analysis results (Wilcoxon’s test for pairs associated with the Bonferroni correction)

Whereas the analysis of individual components of the scale showed that:

- the smallest distance in the macro-social public sphere, the respondents showed towards the Orthodox, and then towards the Protestants and SDA, Jews, Jehovah’s Witnesses, Buddhists, and the largest towards Hindus and Muslims.
- the smallest distance in the micro-social public sphere the respondents showed towards the Orthodox, and then towards the Protestants, SDA, Jehovah’s Witnesses, and the largest towards Muslims, Hindus, Buddhists, and Jews.
- in the private sphere, respondents showed:
- Smaller distance towards Orthodox than towards people of other religions
- Smaller distance towards Protestants than towards people of other religions, except for Orthodox (they were perceived more favorably) and Jews (no significant differences).
- Then, a smaller distance was showed towards SDA and Jews, Jehovah’s Witnesses, Buddhists, and the largest towards Muslims and Hindus.
- the smallest distance in the intimate sphere the respondents showed towards the Orthodox - only they obtained scores above the mid-point of the scale. The largest distance was noted towards Muslims, and then towards Hindus, Buddhists, Jehovah’s Witnesses, Protestants, SDA, and Jews.
- the smallest distance in the biological sphere the respondents showed towards the Orthodox and then the Protestants, Jews and SDA, Jews, Jehovah’s Witnesses, Buddhists, Hindus, and the largest towards Muslims (Table 2).

Table 2 Social distance of the surveyed nurses towards certain religious groups.

#### Social dominance orientation analysis

In the study, the Social Dominance Orientation (SDO) scale was used to diagnose the level of acceptance of inequalities between groups. The scale allows defining attitudes towards foreign groups, especially minority ones, and therefore it is a good instrument for assessing xenophobic, authoritarian, and racist attitudes [6].

A detailed analysis of the Social Dominance Orientation in the study group is presented in the article “Attitudes of Polish Nurses Toward Selected National Groups” [5]. However, it should be noted that in the presented studies, the respondents in all 3 subscales SDO on average scored below the mid-point of the scale (3), therefore showing the low level of violence (mean 1.82), oppressiveness (mean 2.46), and anti-egalitarianism (mean 2.24).

Statistical analysis showed a relationship between Social Dominance Orientation and the modified in the study Bogardus Scale. All subscales of the SDO significantly and negatively correlated with attitudes towards each religion (*p* < 0,05), the higher: orientation on violence; orientation on oppressiveness; orientation on anti-egalitarianism – the larger the social distance towards representatives of each religion (Table 3).

**Table 3:** The results of Social Dominance Orientation in the surveyed group of nurses

Religion	Correlation of the Bogardus Scale with the violence subscale			
	Correlation coefficient	p *	Dependence direction	Dependence strength
Jews	−0,184	*p* < 0,001 NP	negative	very weak
Orthodox	−0,17	*p* < 0,001 NP	negative	very weak
Protestants	−0,184	*p* < 0,001 NP	negative	very weak
Seventh-day Adventists	−0,174	*p* < 0,001 NP	negative	very weak
Jehovah’s Witnesses	−0,194	*p* < 0,001 NP	negative	very weak
Muslims	−0,157	*p* < 0,001 NP	negative	very weak
Hindus	−0,185	*p* < 0,001 NP	negative	very weak
Buddhists	−0,172	*p* < 0,001 NP	negative	very weak
Religion	Correlation of the Bogardus Scale with the violence subscale			
	Correlation coefficient	p *	Dependence direction	Dependence strength
Jews	−0,109	*p* < 0,001 NP	negative	very weak
Orthodox	−0,095	*p* = 0,001 NP	negative	very weak
Protestants	− 0,108	*p* < 0,001 NP	negative	very weak
Seventh-day Adventists	−0,1	*p* < 0,001 NP	negative	very weak
Jehovah’s Witnesses	−0,112	*p* < 0,001 NP	negative	very weak
Muslims	−0,121	*p* < 0,001 NP	negative	very weak
Hindus	−0,123	*p* < 0,001 NP	negative	very weak
Buddhists	−0,122	*p* < 0,001 NP	negative	very weak
Religion	Correlation of the Bogardus Scale with the violence subscale			
	Correlation coefficient	p *	Dependence direction	Dependence strength
Jews	−0,163	*p* < 0,001 NP	negative	very weak
Orthodox	−0,173	*p* < 0,001 NP	negative	very weak
Protestants	−0,151	*p* < 0,001 NP	negative	very weak
Seventh-day Adventists	−0,178	*p* < 0,001 NP	negative	very weak
Jehovah’s Witnesses	− 0,148	*p* < 0,001 NP	negative	very weak
Muslims	−0,115	*p* < 0,001 NP	negative	very weak
Hindus	−0,186	*p* < 0,001 NP	negative	very weak
Buddhists	−0,168	*p* < 0,001 NP	negative	very weak

*P = Normal distribution of both correlated variables, Pearson’s correlation coefficient; NP = Lack normality of distribution of at least one of the correlated variables, Spearman’s correlation coefficient

The analysis of the impact of selected variables on attitudes towards representatives of certain religion groups showed that attitudes significantly (*p* < 0.05) correlate with the following:

- Age – with the exception of Muslims – the older respondent, the larger the distance.
- Seniority in the profession- except for Muslims - the longer the seniority, the larger the distance.
- Frequency of having contact with different religions - the more frequent contact, the smaller the social distance towards its followers.
- Education - the higher the level of education, the smaller the distance towards other religions. People with master’s degrees showed a smaller distance towards Muslims than other people.
- Place of residence - the larger the city, the smaller the distance towards the Buddhists; people from cities with more than 20,000 inhabitants had a smaller distance towards Jehovah’s Witnesses; people from cities with over 20,000 inhabitants has a smaller distance towards other religions than people from cities with less than 20,000 inhabitants and the villages.
- Living in another country - people who lived abroad in the past had a smaller distance towards the Jews, Protestants, and Muslims.
- Providing healthcare (in the past or at the moment) for a person of other religion - people with such experiences showed a smaller social distance towards the representatives of each religion, with the exception of Muslims.
- Participation in religious practices - people participating in religious practices less than once a week showed a smaller distance towards representatives of other religions than people who participated in such practices once or several times a week.
- Religiousness - the more religious the respondent was, the larger the social distance he had towards Muslims, Hindus, and Buddhists; atheists and those who answered “hard to say” showed a smaller distance towards the representatives of other religions than those who were “rather deeply” and “deeply” religious.
- Participation in cultural events related to representatives of other religions - only towards Jews, Orthodox, Protestants, and SDA – people participating in such events showed a smaller distance towards the representatives of these religions.
- Participation in training on transcultural nursing - people who participated in such training in the past showed a smaller social distance towards representatives of each religion.

Attitudes did not correlate significantly (*p* > 0.05) with specialization in nursing, gender, and religion of respondents (however, the results should be treated with caution, as only 32 men, 17 atheists, and 7 non-Catholics participated in the study).

## Discussion

The group of nurses who participated in the study reliably represents the population of nurses in Poland, where the average age of nurses is equal to 50.79 years (in own studies 46.13 ± 10.09 years of age), the majority, i.e., 97.93% of nurses are women (in own studies 96.7%), and 13.69% of nurses have specialization (in own studies 16.44%) most often having secondary vocational education [12]. The overwhelming majority, i.e., 92.29% of respondents were Catholics, while according to the National Census of 2011 [1], 87.7% of Polish society declared themselves as Catholics. Therefore, it can be assumed that the results concerning the social distance of nurses from Lesser Poland towards representatives of different religions will also apply to nurses in Poland. In recent years, the interest in cultural competences in medicine has increased. There are many articles that significantly contribute to knowledge on this matter, unfortunately, not referring to the attitudes of health care workers/nurses towards representatives of different cultures.

In order to find the results of other researchers for the discussion, keywords: nurses, attitudes, religion, social distance, domination orientation were entered (without time limit) into three reference databases MEDLINE - PubMed, Scopus, and Google Scholar. The titles and abstracts were checked, but no articles that could be used in the discussion were found. Due to the inability to compare attitudes of Polish nurses with attitudes towards representatives of other religions of nurses of other nationalities, it was decided to compare the results obtained by the CBOS [13] surveys of Polish society. This comparison will allow deducing whether the occupational group of nurses differs in terms of attitudes towards Others from Polish society. However, it should be noted that this comparison is subject to certain limitations. First, the CBOS research concerned only attitudes towards Muslims, Orthodox, Jehovah’s Witnesses, Protestants, Jews, and Buddhists. Second, there were no average results obtained, but only a percentage of answers to specific questions. Third, the Bogardus scale, modified to four items, was used. The scale used in the CBOS survey included the following questions: What would your attitude be towards .... (name of religion) was your colleague at work; the nearest neighbor; employer; daughter-in-law or son-in-law. This corresponds to the modified Bogardus scale applied in the own research in the sphere of distance in the micro-social public sphere (accepting Other as a co-worker, neighbor, employer) and distance in the intimate sphere (accepting that family member of the respondent is in a relationship with Other).

In the presented studies, the nurses showed moderate social distance towards representatives of other religions. The social distance above the mid-point of the Bogardus scale, indicating a small predominance of positive and neutral attitudes over the negative ones, was noted towards the Orthodox, Protestants, SDA, Jehovah’s Witnesses, and Jews. On the other hand, the distance below the mid-point of the scale, indicating a small predominance of negative and neutral attitudes over positive ones, was noted towards Muslims, Buddhists, and Hindus. In the micro-social public sphere, the respondents showed the smallest distance towards the Orthodox, and then towards the Protestants, SDA, Jehovah’s Witnesses, and the largest towards Muslims, Hindus, Buddhists, and Jews. The majority of adult Poles surveyed by CBOS [13] would have no objection that people of different religions would work with them or would be their closest neighbors. Likewise, it would generally be accepted for the followers of other religions to be their superiors. In the intimate sphere, the smallest distance was showed by nurses that we surveyed towards the Orthodox - only Orthodox obtained results above the mid-point of the scale. The largest distance was noted towards Muslims, then Hindus, Buddhists, Jehovah’s Witnesses, Protestants, SDA, and Jews. Also, for those surveyed by CBOS [13], the most difficult to accept would be the marriage of one’s own child with a person of a different religion. Also, almost three-quarters of the respondents would have no objections if their son or daughter would marry a follower of the Orthodox Church. The majority of respondents would also accept the marriage of their child with a Protestant (61%) or a follower of Judaism (58%). Almost half of the respondents (49%) would have no objection to having a Buddhist for a daughter-in-law or son-in-law. Considerable controversy was for their child to be in a relationship with Jehovah’s Witness (46%) and Muslim (40%). It seems that attitudes towards representatives of other religions of the Polish society are better than the attitudes of the surveyed nurses.

As a result of the religious homogeneity of Polish society, Poles often do not know the followers of other religions and religious associations in person. The only religious association whose members are known by most of the Poles in person are Jehovah’s Witnesses (60%). Less than one-third of the respondents know an Orthodox, and a little bit more than one-fifth a Protestant. When it comes to followers of Judaism and Islam, it was 13 and 8% for followers of Buddhism [13]. Similarly, in the presented study, surveyed nurses had most frequent private or professional contact with Jehovah’s Witnesses - 89.27% and the followers of Orthodox Church - 56.45%; followers of Judaism - 43.62%; Protestants - 41.77%; Muslims - 30.11%, and Buddhists - 20.36%.

The own research shows that there is a significant dependency when it comes to the level of social distance of respondents on age, seniority in the profession, place of residence, level of education, frequency of having contact with people of different religions, living and working abroad in the past, providing healthcare services for a person of other religion, participation in religious practices, religiousness, participation in training on transcultural nursing. CBOS [13] surveys showed that the attitudes of the respondents differentiate: towards Orthodox – age and contact; towards Protestants – the level of education, frequency of participation in religious practices and knowing follower of Protestantism in person; towards the followers of Judaism – the level of education; towards Muslims - age; towards Buddhists – age, and level of education; towards Jehovah’s Witnesses - contact and frequency of participation in religious practices.

According to the results of the report on Social Diagnosis 2011 [14], nurses that we surveyed are not a representative group for Polish society, as the own research, based on the SDO showed that the majority of respondents had a low violence score (85.75%), anti-egalitarianism (83.04%) and oppressiveness (69.28%). On the other hand, the results of the Social Diagnosis 2011 stated that 79% of respondents showed an egalitarian attitude and 51% dominant attitude. However, the comparison of the results obtained by the authors with the results of Social Diagnosis 2011 should be treated with caution, as the authors of the Social Diagnosis used only 4 questions of the SDO and made their conclusions on their basis.

Although the attitudes of Polish nurses were not compared with the attitudes of nurses from other countries towards representatives of other religions, the literature review by Van der Kluit and Goossens (2011) provides information on the factors that may influence the attitudes of nurses towards patients [15].

Parrillo and Donoghue (2005, 2013), while studying American students, showed that the average level of social distance towards all ethnic groups and the gap between the groups with the highest and the lowest levels of distance decreased compared to previous studies. Moreover, it has been shown that gender, race, and country of origin are significant determinants when it comes to the level of social distance towards all groups [16, 17].

The Segev, Mor, Even-Zahav (2020) study analyzed cultural intelligence (CQ) and social distance among students (of nursing, social work, behavioral sciences) from the majority (Jewish) and minority (Arab) groups. The results revealed a negative relationship between CQ and social distance. Higher CQ was found among students from the minority group. It found that students from the minority group were more open to intercultural exchanges [18]. Hadar-Shoval, Alon-Tirosh, and Morag (2019) revealed less negative stereotypes among Jewish nursing students than among Arab nursing students. Nevertheless, Jewish students were less likely to reduce social distances. They found no differences between students starting and in the process of studying in terms of stereotypes. However, the advanced students expressed a greater desire to reduce the social distance than the beginning students. The researchers concluded that the perception of social relationships is influenced by different contexts, including the specific context of students (studying together and working together in the future) and the broader overall context of relationships between groups. The results of their research indicate that programs aimed at developing cultural sensitivity and improving relationships in a divided society should provide different answers for each group and focus on the willingness to cooperate and reduce social distance rather than on trying to eliminate stereotypes [19].

The study by Nyaupane, Timothy, and Poudel (2015) analyzes the role of social distance in relations between people of different faiths visiting the holy place of Buddha’s birth. The result of this study suggests that Hindus and Christians visited the site because they considered the Buddhas more concerned with their faith than other groups. Moreover, this article shows that people perceive themselves as tourists, pilgrims, tourists and pilgrims, or as none of these groups. The research indicates that those who identify as pilgrims have higher religious motivations, and those who identify as tourists have higher recreational or cultural motivations. In this study, a social distance determined relational structures, similarities and differences between travelers of different religions using the same tourist areas. Belonging to groups is one of the elements of identity. However, while we may choose some groups to which we should belong, categories such as race and ethnicity - along with gender and religion to some extent - are something we cannot choose [20]. The analysis of the results of the research by Rajagukguk et al. (2018) allowed to conclude that the social distance in religiosity and empathy is negatively correlated [21].

Ghanean, Nojomi and Jacobsson, 2014 conducted research to assess attitudes towards mentally ill patients from Islamic countries. It has been shown that the level of negative attitudes in Iran was similar to that in other countries and cultures, although taking into account the fact that illness, including mental illness, is the result of Allah’s will, it could suggest less stigmatization of people with mental disorders [22].

Negative perceptions and distancing attitudes towards people with mental illnesses and victims of sexual violence are common in societies and cultures of Sub-Saharan Arica, for example, among students from Nigeria [23, 24]. The study by Akinbobol, Zugwaj (2019) examined the relationship between emotional empathy, social distance, and the attitude towards mental illness of police officers who contacted mentally ill people during their service. It turned out that emotional empathy and its dimensions, as well as social distance, had a significant impact on the attitude towards people with mental illnesses [24].

Adegboyega et al. (2021) started a discussion on social distance and its socio-behavioral consequences at the time of COVID-19. Social distancing has had positive effects on religious contacts, such as reducing the spread of the disease among the faithful and enabling the use of technological innovations. Conversely, it negatively affected social cohesion and left its mark on religious practice. It should be suspected that it may also influence the shaping of attitudes towards followers of various religions, which would undoubtedly be worth examining [25].

In the opinion of the authors of the manuscript, as in the opinion of Feliksiak [26], the attitudes towards the followers of other religions that function in Poland in society and among nurses are largely mediated, i.e. they are based mainly on information obtained from the media, and to a small extent on the contact with the followers of a particular religion. These contacts may take place sporadically, usually during trips abroad or during integration meetings. The attitude is also fostered by the associations of particular religions or stereotypes functioning in a society that occur in a significant part of the respondents. Personal acquaintance with a follower of a different religion may not thaw the relations but may also make the religion or, more broadly, culture perceived less critically.

## Conclusions

1. The respondents showed the smallest social distance towards the Orthodox and Protestants, and the largest towards the Muslims.
2. There is a significant dependency when it comes to the level of social distance of respondents on age, seniority in the profession, place of residence, level of education, frequency of having contact with people of different religions, living and working abroad in the past, providing healthcare services for a person of other religion, participation in religious practices, religiousness, participation in training on transcultural nursing.
3. The relationship between SDO and the modified Bogardus Scale has been demonstrated. All subscales of the SDO were significantly and negatively correlated with the Bogardus scale.

## Data Availability

The datasets used and analyzed during the current study are available from the corresponding author on reasonable request.
